# Investigation of Suitable Detection Angles for Carbon-Ion Radiotherapy Monitoring in Depth by Means of Secondary-Ion Tracking

**DOI:** 10.3389/fonc.2021.780221

**Published:** 2021-11-29

**Authors:** Laura Ghesquière-Diérickx, Annika Schlechter, Renato Félix-Bautista, Tim Gehrke, Gernot Echner, Laurent Kelleter, Mária Martišíková

**Affiliations:** ^1^ Department of Medical Physics in Radiation Oncology, German Cancer Research Center (DKFZ), Heidelberg, Germany; ^2^ National Center for Radiation Research in Oncology (NCRO), Heidelberg Institute for Radiation Oncology (HIRO), Heidelberg, Germany; ^3^ Heidelberg Medical Faculty, University of Heidelberg, Heidelberg, Germany; ^4^ Faculty of Physics and Astronomy, Heidelberg University, Heidelberg, Germany

**Keywords:** carbon-ion radiotherapy, *in-vivo* treatment monitoring, inter-fractional anatomical changes, secondary-ion tracking, beam fragmentation, silicon pixel detector, Timepix3

## Abstract

The dose conformity of carbon-ion beam radiotherapy, which allows the reduction of the dose deposition in healthy tissue and the escalation of the dose to the tumor, is associated with a high sensitivity to anatomical changes during and between treatment irradiations. Thus, the monitoring of inter-fractional anatomical changes is crucial to ensure the dose conformity, to potentially reduce the size of the safety margins around the tumor and ultimately to reduce the irradiation of healthy tissue. To do so, monitoring methods of carbon-ion radiotherapy in depth using secondary-ion tracking are being investigated. In this work, the detection and localization of a small air cavity of 2 mm thickness were investigated at different detection angles of the mini-tracker relative to the beam axis. The experiments were conducted with a PMMA head phantom at the Heidelberg Ion-Beam Therapy Center (HIT) in Germany. In a clinic-like irradiation of a single field of 3 Gy (RBE), secondary-ion emission profiles were measured by a 2 cm^2^ mini-tracker composed of two silicon pixel detectors. Two positions of the cavity in the head phantom were studied: in front and in the middle of the tumor volume. The significance of the cavity detection was found to be increased at smaller detection angles, while the accuracy of the cavity localization was improved at larger detection angles. Detection angles of 20° – 30° were found to be a good compromise for accessing both, the detectability and the position of the air cavity along the depth in the head of a patient.

## 1 Introduction

There are several advantages of carbon ion radiotherapy (CIRT) over photon and proton RT. The narrow depth-dose deposition profile of carbon ions, as well as their higher LET and RBE compared to protons or photons can be used to deliver more conformal dose distributions to the tumor volume while sparing nearby organs at risk (1). However, the CIRT dose distribution is very sensitive to treatment geometry variations such as anatomical changes or changes in the patient positioning. Those variations can lead to severe under-dosage of the tumor or over-dosage of healthy tissue (2). On-line and *in-vivo* monitoring methods of the dose distribution could allow potential inter-fractional changes to be detected, offering to make CIRT safer and more effective (3, 4).

Most of the proposed CIRT monitoring methods exploit the information on the dose distribution in the patient carried by products of nuclear interactions of the carbon-ion beam with the patient tissue. These products can be annihilation photons from β+ emitters, prompt gamma rays or prompt charged fragments (also known as secondary ions) (5). The developed CIRT monitoring methods use the distribution of these interaction products to detect the position of the primary carbon-ion beam in the patient. Even in the case when the correlation between nuclear products and dose is low, the measured nuclear product distributions of different treatment fractions can be directly compared to each other (or to Monte Carlo simulations) in order to draw conclusions on potential treatment variations (6).

This contribution investigates the secondary-ions-based method. It aims to quantify the influence of the detection angle on the performance of the cavity detection within a head phantom. As the secondary ion production is forward-peaked, i.e. they are mainly emitted in the direction of the primary carbon-ion beam, experimental configurations of secondary-ion-based monitoring methods use forward detection angles *α* with respect to the beam direction. Over the years, secondary-ions detection angles ranging from 0° (7, 8) to 90° (9) were investigated. Most of the recently published experiments have been measured at either 30° (10, 11), or at 60° and 90° (12–14). The first results of a clinical study were taken at a composite detection angle of 60° (horizontal plane) and 30° (vertical plane) relative to the beam direction (15). However, no systematic investigation of the influence of the detection angle on the performance of the detection system in a realistic clinical setting has been published to this date.

In this contribution, the efficiency of our secondary-ion-based monitoring system to detect a 2-mm-thick air cavity in a head phantom is investigated for different detection angles. Two metrics are used to identify the optimal detection angle for this specific set-up: the detectability of the changes induced by the air cavity, and the localization of the change along the depth in the head phantom. The detectability is quantified as a metric based on the measured deviation in the detected charged fragments. The localization is defined as the reconstructed cavity position. Additionally, the robustness of the method is investigated for two different cavity positions along the depth in the head phantom.

Detection angles from 10° to 50° in steps of 10° are investigated. The detectability is expected to improve with larger number of detected secondary ion tracks, i.e. to improve at smaller angles, while the localization is expected to improve with the spatial resolution, thus to be better at larger angles. This is because the spatial resolution is determined by Multiple Coulomb Scattering (MCS) of the fragments in the phantom (16, 17), which is projected onto the beams axis under the detection angle (12). However, the optimal angle for the detection of the secondary ion tracks is currently unknown.

## 2 Materials and Methods

### 2.1 The Heidelberg Ion-Beam Therapy Center

The experiments were carried out at the Heidelberg Ion-Beam Therapy Center (HIT) in Germany (18, 19). At the HIT facility, carbon-ion irradiation can be performed in four rooms - three treatment rooms and one experimental room. Two treatment rooms have fixed horizontal beam lines while the third treatment room is equipped with a 360° revolving carbon-ion gantry. The experimental room, which is used for quality assurance and research, houses a horizontal beam line that is identical to those in the treatment rooms. The measurements of this study were performed in the experimental room. HIT offers 255 discrete energy steps ranging from 88.83 MeV/u to 430.10 MeV/u for carbon ions. These energy steps correspond to a range of penetration depths in water of 2 cm to 30 cm (20) with step sizes of 1 mm to 1.5 mm and adjustable beam sizes in 6 steps ranging from 3.4 mm to 13.4 mm FWHM.

To cover the three-dimensional tumor volume with the required dose, the HIT facility uses an active raster scanning system (20). This system involves the separation of the target volume into iso-energy slices (IES) that are irradiated slice-by-slice. The manipulation of the lateral beam spot position is performed with a magnetic scanning system. The beam nozzle includes a ripple filter for dose flattening in the spread-out Bragg peak and a beam application monitor system (BAMS), which registers the lateral pencil beam position, size and number of particles for each pencil-beam spot (21).

### 2.2 Head Phantom

For this work, a homogenous Poly(methyl methacrylate) (PMMA) cylinder was used as a surrogate for a patient head. The cylinder has a height of 90 mm and a diameter of 160 mm. At its center there is a cubic opening of 80 × 80 × 80 mm^3^ that could be filled with PMMA slabs as shown in **Figure 2**. The relative stopping power (RSP) of the head phantom, measured with a PTW Peakfinder Water Column (T34080 Bragg Peak chamber and TANDEM XDR electrometer) was found to be 1.163 for the cylinder and 1.151 for the PMMA slabs, both were thus comparable to the RSP of soft human tissues (22).

### 2.3 Treatment Plan

Using a clinical CT scanner (SIEMENS Sensation Open device) and the corresponding clinical imaging protocol for head patients, a CT image of the head phantom was acquired. Based on that, a CIRT treatment was designed using the Siemens syngo RT Planning system (Siemens Healthcare GmbH, Erlangen, Germany). The treatment plan targets a virtual spherical tumor (volume of 70.06 cm^3^) centered in head phantom with a fraction dose of 3 Gy (RBE), comparable to a realistic clinic-like single-field CIRT fraction dose. The total number of primary carbon ions was 5.69 × 10^8^, distributed over 8356 raster points (scan grid distances of 2.0 mm × 2.0 mm), divided into 19 iso-energy slices (step width of 3.0 mm range in water) ranging from 167.66 to 239.45 MeV/u and with a beam spot size in air of 6 mm (FWHM).

### 2.4 Mini-Tracker

**Figure 1:**
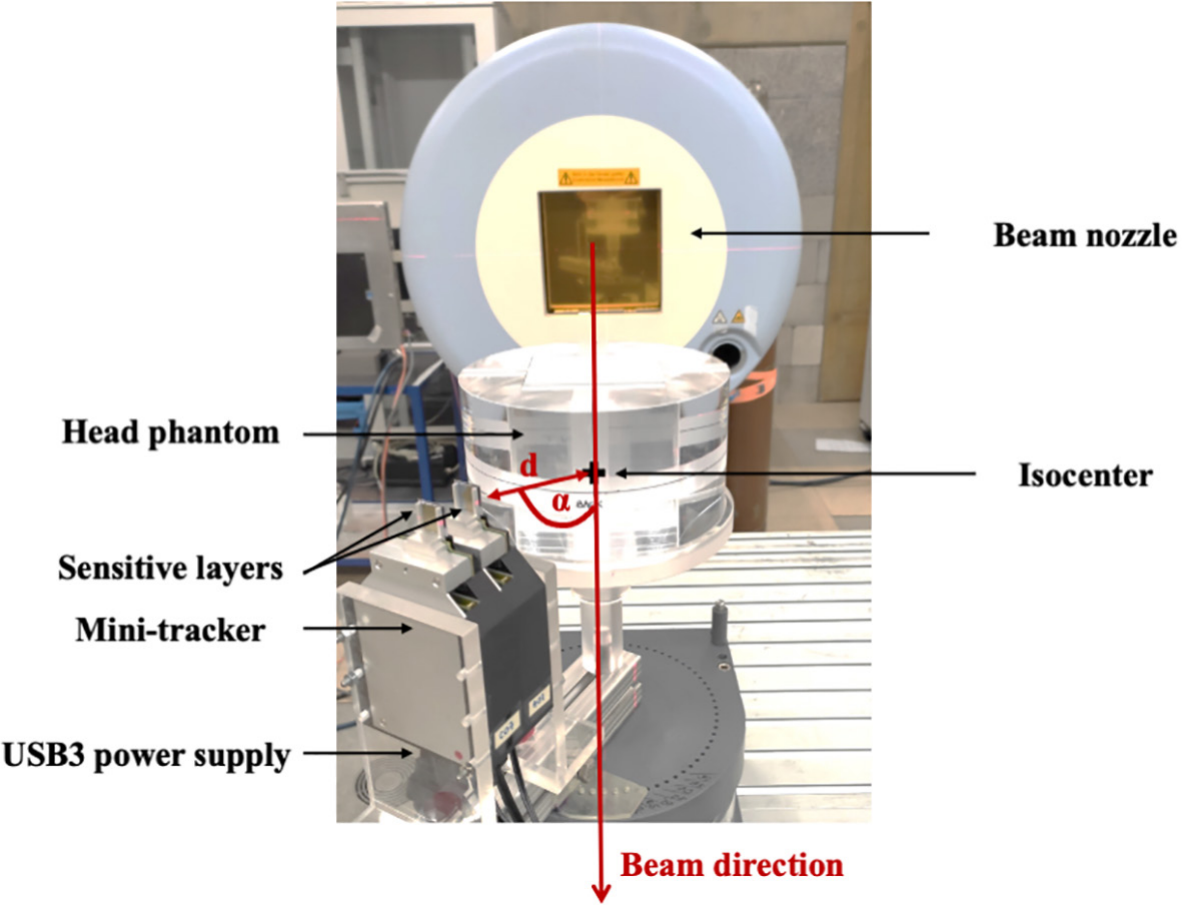
A head phantom composed of PMMA was irradiated with a clinic-like carbon-ion treatment field. Charged fragments were detected by a mini-tracker based on two Timepix3 chips. Several positions of the mini-tracker, characterized by the distance d between the front detector and the isocenter as well as the angle α with respect to the beam direction, were investigated.

**Figure 2:**
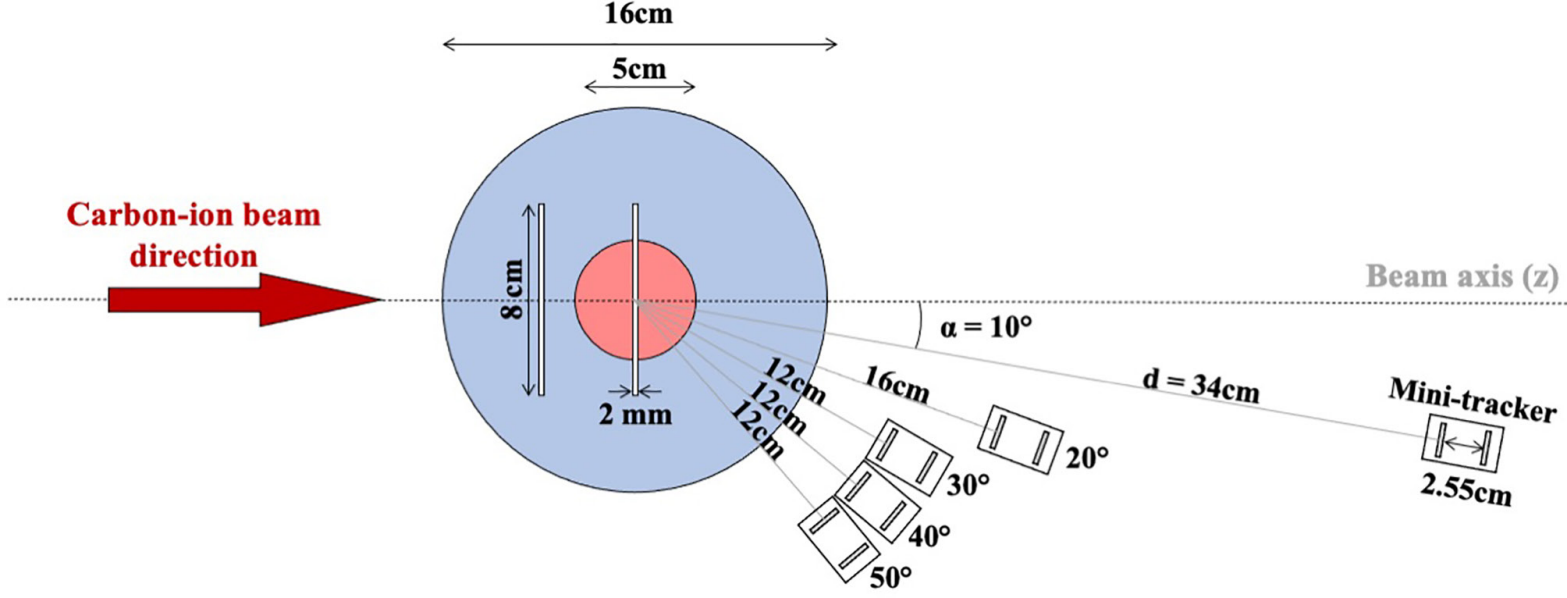
Schematic top view of the experimental set-up. The blue circle represents the cylindrical head phantom and the red circle represents the irradiated spherical tumor volume. The vertical white bars represent the positions of the air cavity (either upstream of the tumor volume or at the isocenter). The distance between the room isocenter and the front detector of the mini-tracker is denoted as d and α is the angle of the mini-tracker axis with respect to the axis of the treatment field direction (beam axis). The following mini-tracker positions were investigated: 10° at a distance d of 34 cm, 20° at a distance of 16 cm, and 30°, 40°, and 50° at a distance of 12 cm.

A mini-tracker (see **Figures 1**, **2**) made of two pixelated detectors was used to detect and track individual charged nuclear fragments. Each of the employed AdvaPIX TPX3 modules was equipped with a Timepix3 chip based on the hybrid semiconductor pixel detector technology developed within the Medipix3 Collaboration at CERN (23). The sensitive layer of each detector is 300 μm thick crystalline silicon with an area of 14 × 14 mm² divided into 256 × 256 pixels (pixel pitch of 55 μm). The time resolution of a single TPX3 module is 1.56 ns. The sensors were operated at a bias voltage of 10 V. This relatively low bias voltage causes a partial depletion of the silicon layer, which leads to a larger cluster size and thus enables an more precise hit position measurement based on the calculation of the energy-weighted center of mass of the cluster (24). The energy threshold was set to 3 keV, ensuring a noise-free data acquisition.

The distance between the two sensitive silicon layers was set to 25.5 mm (see **Figure 2**). The two TPX3 modules were connected *via* a synchronization cable and to a mini PC notebook *via* two USB 3.0 cables (see **Figure 1**). The detector settings and the data acquisition were controlled using the Pixet software (version 1.6.5.778) (25).

### 2.5 Experimental Set-Up

The following experimental set-up (see **Figure 1**) was used to measure individual secondary-ion tracks with a mini-tracker placed at different detection angles. The center of the head phantom, which was identical to the center of the tumor, was aligned to coincide with the isocenter of the experimental room. Individual secondary ions emerging from the head phantom were tracked by the mini-tracker placed behind the phantom at different positions defined by the detection angle α (10°, 20°, 30°, 40°, 50°) with respect to the beam direction and the distance *d* (12 cm, 16 cm and 34 cm, depending of the detection angle) to the isocenter, as illustrated in **Figure 2**. The mini-tracker axis was made to point at the isocenter in order to cover the region before and behind the tumor in the field-of-view. The standard distance from the front detector to the isocenter was 12 cm. In order to cope with the signal pile-up seen at smaller detection angles where the fluence rate increases rapidly, the mini-tracker module was positioned further away from the isocenter.

To mimic inter-fractional anatomical changes, a 2 × 80 × 80 mm^3^ PMMA slab in the head phantom was removed, creating an air cavity as indicated by the white slabs in the drawing of the head phantom (**Figure 2**). In this study, two different cavity positions were investigated: upstream of the tumor volume (at a depth of -40 mm relative to the isocenter) and at the isocenter (at a depth of 0 mm). Only a single air-cavity position was measured at a time.

Four kinds of measurements, corresponding to four treatment fractions (one reference fraction and three follow-up fractions), were performed at each investigated angle. The first fraction was defined as the reference fraction. The second fraction was a repetition of the first fraction, in order to investigate fraction-to-fraction variation of the signal in case that there are no internal geometry changes. In the third and fourth fractions, the air cavity was inserted at a depth of -40 and 0 mm, respectively. Mini-tracker and head phantom were not moved between measurements of different air-cavity positions.

In order to simulate a future detector upgrade with ≥8 mini-trackers, each measurement was repeated eight times and the obtained data was summed up. A larger detection area can be approximated by a repetition of the measurement because of the small size of the mini-tracker and the resulting small angular variance. The angular variance could be further reduced by arranging the mini-trackers on a circle with a constant detection angle relative to the beam axis, thus utilizing the angular symmetry of the fragment field.

### 2.6 Data Acquisition

For each of the two Timepix3 detectors in the mini-tracker, the measured raw data consist of a stream of pixel hits with the time of arrival (ToA) and the time over threshold (ToT) being recorded. Using in-house written Matlab^1^ and C++ routines, the raw data was post-processed as follows: for each measured secondary ion hit, a so-called cluster was formed from the stream of measured pixels. Neighboring pixel signals within ±75 ns were grouped together as a cluster. The cluster size was defined as the number of pixels contained in a cluster, the cluster arrival time was defined as the minimum ToA of any pixel in the cluster. Clusters with a size of one single pixel were excluded from further data processing, as those were expected to represent noise or background radiation (photons or electrons) (26).

### 2.7 Data Analysis

#### 2.7.1 Secondary-Ion Tracks and Emission Profiles

Coincident clusters measured within ±75 ns in both mini-tracker sensor layers were considered as hits caused by the same secondary ion. The straight line connecting the energy-weighted centers of mass of the coincident clusters in the two sensor layers was defined as a secondary ion track (26).

To approximate the origin of a secondary ion in the head phantom (the fragmentation vertex), a three-dimensional pencil-beam-based back-projection method was used (27). This back-projection method finds the line that represents the shortest distance between the extrapolated secondary ion track and the beam axis of the respective carbon-ion pencil beam at the time of the track detection. The middle point on this connecting line is defined as the origin of the secondary ion.

The angular projection uncertainty is a function of the uncertainty of the hit position and the distance between the detection layers. The uncertainty of the hit position was approximated as that of a uniform distribution in a pixel. On top of that comes the MCS angle in the front detector (1 mm Silicon), resulting in an estimated angular projection uncertainty of 0.072°+0.069°=0.141°. For comparison, the 1/e MCS angle of 200-MeV-protons travelling through 8 cm of PMMA is approximately 0.84° (28).

The histogram of the fragment origins along the beam axis is referred to as a secondary-ion (or fragment) emission profile. A bin size of 5 mm was used as a reasonable bin size. The effect of the bin size on the data analysis is investigated in section 3.2.4. The number of detected secondary ions in a bin is denoted as *N*, with the uncertainty being the square root of *N* (Poisson statistics).

Subsequently, the measured secondary-ion emission profiles were analyzed with different methods to investigate the impact of the air cavity on the radiation field distribution in the phantom. In particular the detectability and the localization of the air cavity were investigated for different positions of the mini-tracker.

#### 2.7.2 Detectability of the Air Cavity

As the aim of the developed method is to compare the emission profiles of different treatment fractions, the difference between the absolute fragment emission profiles of a follow-up fraction (with or without air cavity) and the reference fraction (without air cavity) was determined using: *Difference* = *N_followUp_* – *N_ref_* with its uncertainty being $\sigma (Difference)=\sqrt{N_{followUp}+N_{ref}}$ where *N_followUp_* and *N_ref_* represent the number of entries in a bin of the follow-up and reference emission profile, respectively.

To quantify the detectability of the inserted air cavity, the integral of the absolute differences along the depth covered by the head phantom was calculated:


$$Integral_{\left|followUp-ref\right|}=\sum_{head\;phantom}\left|N_{followUp}-N_{ref}\right|$$

**Equation 1:** Integral oft the absolute difference of two emission profiles.


$$\sigma (Integral_{\left|followUp-ref\right|})=\sqrt{\sum_{head\;phantom}\left(N_{followUp}+N_{ref}\right)}$$

**Equation 2:** Statistical uncertainty of the integral.

The absolute value allows positive as well as negative deviations to be taken into account. As a consequence, the integral of absolute values will not be equal to zero even in case of no significant deviation. Therefore, it must always be compared to a measurement without cavity in place. The detectability is defined as the number of combined standard deviations of the integral with air cavity above the integral without air cavity. The uncertainty of the detectability is calculated using Gaussian error propagation.


$$Detectability=\frac{Integral_{\left|cavity-ref\right|}-Integral_{\left|noCavity-ref\right|}}{\sqrt{\sigma {\left(Integral_{\left|cavity-ref\right|}\right)}^{2}-\sigma {\left(Integral_{\left|noCavity-ref\right|}\right)}^{2}}}$$

**Equation 3:** Detectability of the air cavity.

#### 2.7.3 Localization of the Air Cavity

In a first step to localize the air cavity, the minimum of the fragment emission profile is determined. Only bins from the start of the head phantom up to the distal end of the tumor volume (depth of -80 mm to +25 mm) are considered when searching for the minimum bin because only anatomical changes in this area can have an impact on the dose distribution.

The location of the air cavity along the depth of the head phantom is defined as the minimum of a second order polynomial drawn through the minimum bin and its two nearest neighbors. The polynomial does not represent a fit since the number of included bins (three) equals the number of degrees of freedom (also three). Instead, the polynomial is used to take into account the information on the minimum location that is included in the direct vicinity of the minimum bin, while simultaneously minimizing the bias from any assumption made on the shape of the minimum that would come with the fit of a function. The uncertainty on the location of the minimum was calculated using Gaussian error propagation.

The suggested procedure for finding the cavity location depends on the bin size since it always takes into account the same number of bins (three), regardless of their size. Therefore, the influence of the bin size on the reconstructed location of the air cavity was investigated by conducting the localization procedure for emission profiles with different bin sizes (see section 3.2.4).

## 3 Results

### 3.1 Secondary-Ion Emission Profiles for Different Mini-Tracker Positions

**Figure 3:**
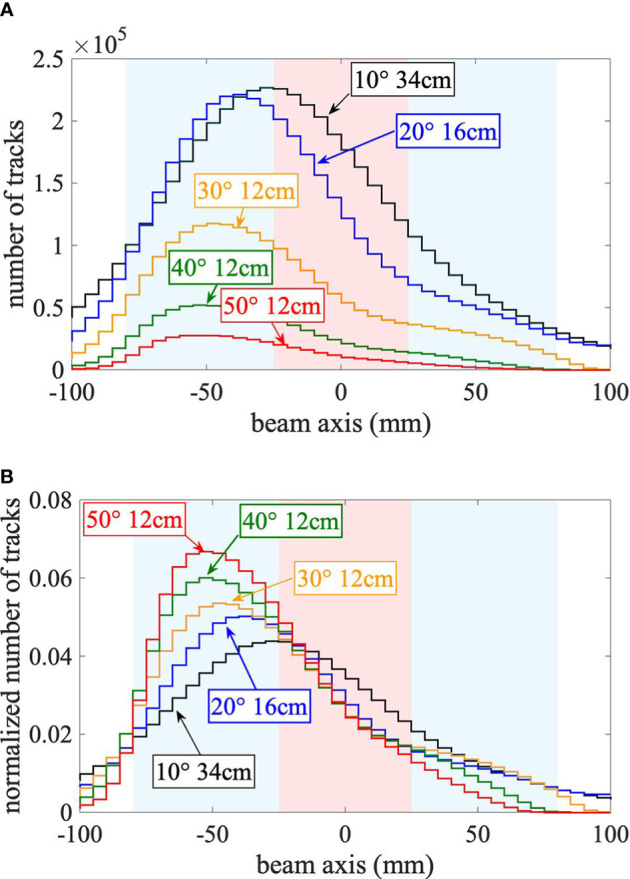
**(A)** Absolute emission profiles. **(B)** Normalized emission profiles. **(A)** Absolute and **(B)** normalized (to the integral) secondary-ion emission profiles along the beam axis for each studied mini-tracker position without air cavity. The cylindrical head phantom (radius of 80 mm) is centered in the isocenter at a depth along the beam axis of 0 mm and is highlighted by the blue area. The spherical target volume (25 mm radius) is represented by the red area. The beam crosses the head phantom from left to right. The statistical uncertainties are plotted as uncertainty bands.

The measured secondary-ion emission profiles were analyzed for each mini-tracker position shown in **Figure 2**. A comparison of the absolute as well as the normalized (to the integral) emission profiles is shown in **Figures 3A**, **B**, respectively. The absolute secondary-ion emission profiles in **Figure 3A** differ mainly in the total number of detected secondary-ion tracks. The number of detected tracks decreases with increasing detection angle, as expected. The relatively small increase in the number of tracks at 10° and 20° results from the larger distance of the mini-tracker to the isocenter, which was chosen in order to technically cope with the higher secondary-ion fluence close to the beam axis.

As visible in **Figure 3B**, due to the geometrical limit of the back-projection method (12), the sharpness of the normalized emission profile increases with increasing detection angle while the effect of Multiple Coulomb Scattering within the phantom, leading to an increased number of tracks that are wrongly back-projected in front of and behind the head phantom, decreases. For large detection angles, the maximum of the emission profile is located at a shallow depth, whereas for small detection angles the maximum is closer to the isocenter. This is a direct geometrical consequence of detection angle: the maximum fragment emission is expected at the entrance to the phantom (highest number and energy of carbon ions and smallest effective detection angle), but the emission profile is smeared out by the projection uncertainty, which increases with decreasing detection angle. The larger distance to the isocenter at small detection angles also contributes to the increase of the projection uncertainty. However, this effect is small in comparison because of the small pixel size of 55 μm.

### 3.2 Impact of an Air Cavity on the Secondary-Ion Emission Profiles For Different Mini-Tracker Positions

In this work, the impact of an inter-fractional change induced by a 2-mm-thick air cavity was analyzed in three ways. First, the change was visualized by calculating the difference between the secondary-ion emission profiles measured with and without the air cavity. In a next step, the detectability and localization of the air cavity were determined.

#### 3.2.1 Secondary-Ion Emission Profiles With and Without Air Cavity

**Figure 4:**
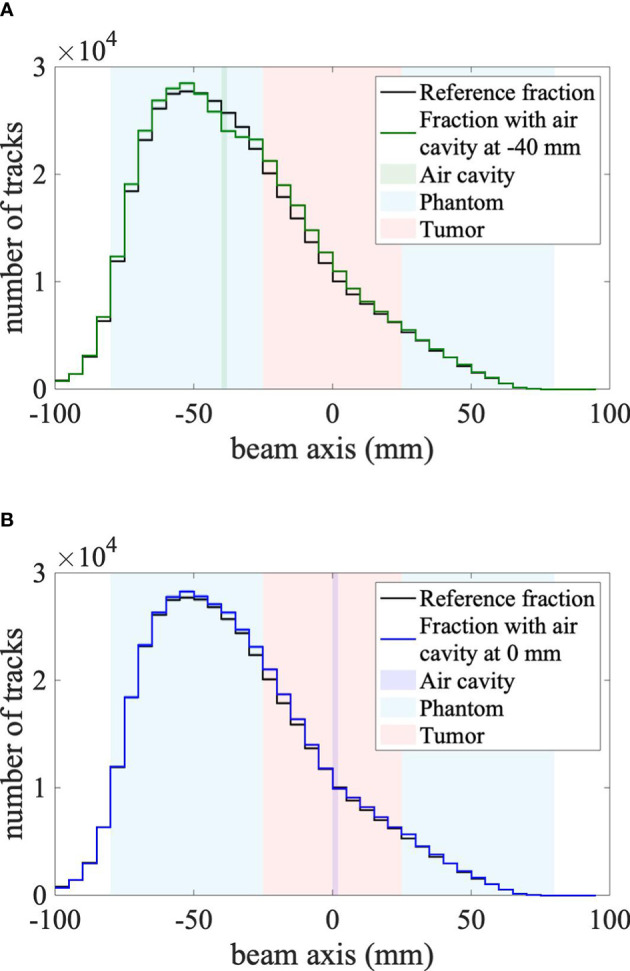
**(A)** Air cavity at -40 mm (in front of the tumor). **(B)** Air cavity at 0 mm (center of the tumor). Secondary-ion emission profiles measured by the mini-tracker placed at an angle of 50° and a distance of 12 cm from the center of the target. The irradiation fraction with a cavity in front of the tumor volume (solid green line) is plotted on the left, whereas the irradiation fraction with a cavity in the middle of the tumor volume (solid blue line) is plotted on the right. The solid black line in both plots represents the reference fraction. Corresponding statistical uncertainties are plotted as uncertainty bands for each profile, which are smaller than the line width. The head phantom is centered at 0 mm and is represented by the blue area, the 25-mm-radius tumor volume is represented by the red area and the inserted air cavity is represented by the vertical green and blue band in **(A, B)**, respectively.

Secondary-ion emission profiles were measured using a mini-tracker placed at five different positions. As an extreme example with the lowest number of measured tracks, **Figure 4** shows the secondary-ion emission profiles for the mini-tracker positioned at an angle 50° and a distance of 120 mm from the isocenter.

**Figure 5:**
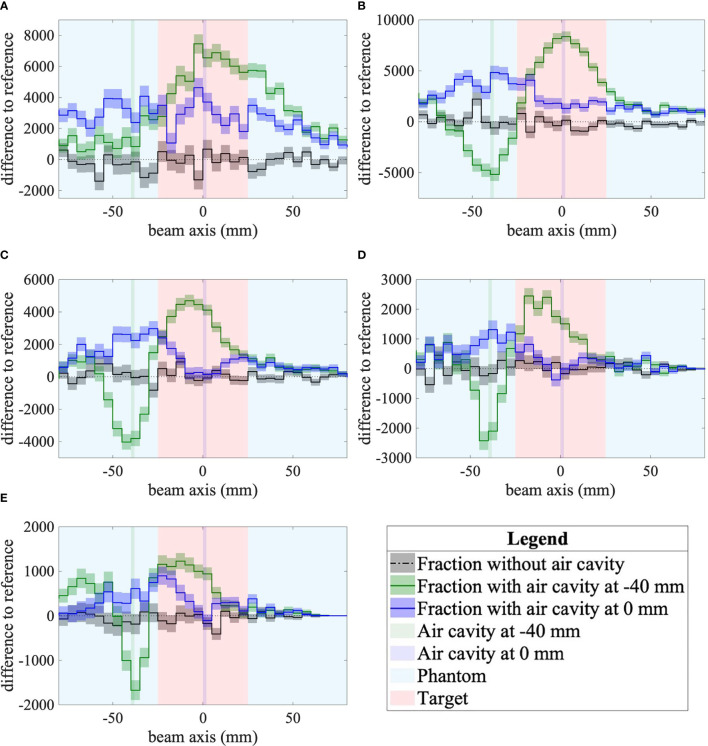
**(A)** α=10°, d=34 cm. **(B)** α=20°, d=16 cm. **(C)** α=30°, d=12 cm. **(D)** α=40°, d=12 cm. **(E)** α=50°, d=12 cm. Differences to the reference of secondary-ion emission profiles for the three follow-up fractions as measured by the mini-tracker placed at different detection angles and distances to the isocenter. The difference between the reference fraction and the fraction without a cavity is shown as a dotted-dashed black line. The difference between the reference fraction and the fraction with a cavity in front of the tumor volume (depth of -40 mm) is plotted in green. The difference between the reference fraction and the fraction with a cavity in the middle of the tumor volume (0 mm) is plotted in blue. The statistical uncertainties are plotted as uncertainty bands for each curve. The plots show the data inside the 80-mm-radius head phantom, which is centered at 0 mm. The red area represents the 25-mm-radius tumor volume and the inserted air cavity is represented by the vertical green and blue bands.

The shape of the measured secondary-ion emission profile is similar for all measured fractions. However, statistically significant differences between the reference irradiation fraction and the fractions with an inserted air cavity are present, particularly when the cavity is placed in front of the tumor volume. Compared to the reference fraction, the secondary-ion emission profile is higher in front of the air cavity, lower at the depth of the cavity and higher behind the cavity. These observations are consistent with (11), due to the expected effect of the air cavity on the primary carbon ions and the charged fragments: the carbon ions penetrate deeper when they cross a low-density region such as the air cavity, leading to a shift of the emission profile towards deeper positions, which explains the larger amount of detected fragments behind the air cavity. In the air cavity itself, fewer fragments are produced, leading to a dip in the secondary-ion emission profile at the depth of the air cavity. Those fragments produced in front of the air cavity have to cross less material in order to reach the mini-tracker, explaining the increased number of detected fragments from shallow depths. For further analysis, the differences between the reference fraction and the follow-up fractions are shown in **Figure 5**.

#### 3.2.2 Difference of Fragment Emission Profiles

For all mini-tracker angles (**Figure 5**), it is observed that the difference between the absolute fragment emission profile without a cavity and the reference (dashed-dotted black curve) is compatible with zero (dotted black line) within its statistical uncertainties, showing the robustness of the method. For both cavity positions and all mini-tracker positions, the difference to the reference measured with an air cavity in place was found to be significantly different from zero, demonstrating qualitatively the detectability even for such a small variation of the phantom geometry.

For the air cavity inserted in front of the tumor, the difference profile reaches a minimum near the depth of the air cavity (-40 mm) for every detection angle except for 10°. The width of this minimum is found to decrease with increasing detection angle, as expected from the projection of the Multiple Coulomb Scattering on the beam axis.

A dip at the depth of the air cavity is visible when the air cavity is located in the isocenter for detection angles of 30° to 50°. It can happen that the dip does not represent an unambiguous minimum. This is partly due to the lower number of fragments of sufficient energy produced at this depth. Moreover, because of the lateral size of the air cavity (80 × 80 mm^2^), fragments that are produced in front of the air cavity and which cross the air cavity on their path to the mini-tracker are less likely to be absorbed because they cross less material. This explains the increased number of detected fragments in front of the air cavity. This effect partly compensates for the dip and makes the identification of the minimum more challenging.

#### 3.2.3 Detectability of the Air Cavity

**Figure 6:**
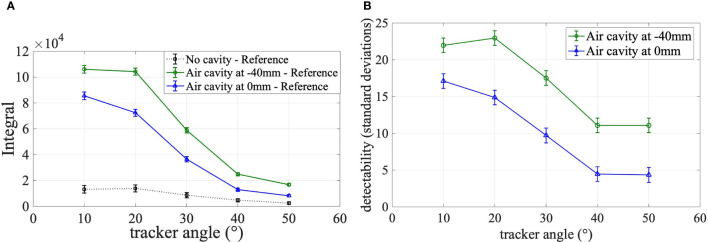
**(A)** Integral of absolute differences, **(B)** Detectability. **(A)** Integral of the absolute difference between secondary-ion emission profiles of the reference fraction and the three follow-up fractions over the target area shown for all investigated mini-tracker positions. The integral of the difference between the reference fraction and the fraction without cavity is plotted as square black markers. The integral values between the reference fraction and the fraction with a cavity in front of the tumor volume are plotted as green circles. The integral between the reference fraction and the fraction with a cavity in the middle of the tumor volume is shown as blue triangles. **(B)** Detectability of the air cavity in units of combined standard deviations.

To quantify the detectability of the air cavity, the integral of the absolute difference between the fraction with/without air cavity and the reference fraction is calculated (**Figure 6A**). At every mini-tracker angle, the integral of the absolute difference between the fraction with an air cavity and the reference fraction (green circles and blue triangles) is significantly higher than the integral of the difference without a cavity (black squares). The integral increases with decreasing detection angle because of the larger number of detected secondary-ion tracks at small angles.

The detectability as defined in Equation 3 is shown in **Figure 6B**. The detectability can also be interpreted as the significance of the measurement. It is observed that the presence of the air cavity is detected for all mini-tracker positions and both air cavity locations with a detectability of more than four combined standard deviations. The detectability is maximized for a detection angle of 20° and 10° for a cavity position of -40 mm and 0 mm, respectively. This can be explained by the number of detected fragment tracks, which is maximized at small detection angles.

#### 3.2.4 Localization of the Air Cavity

**Figure 7:**
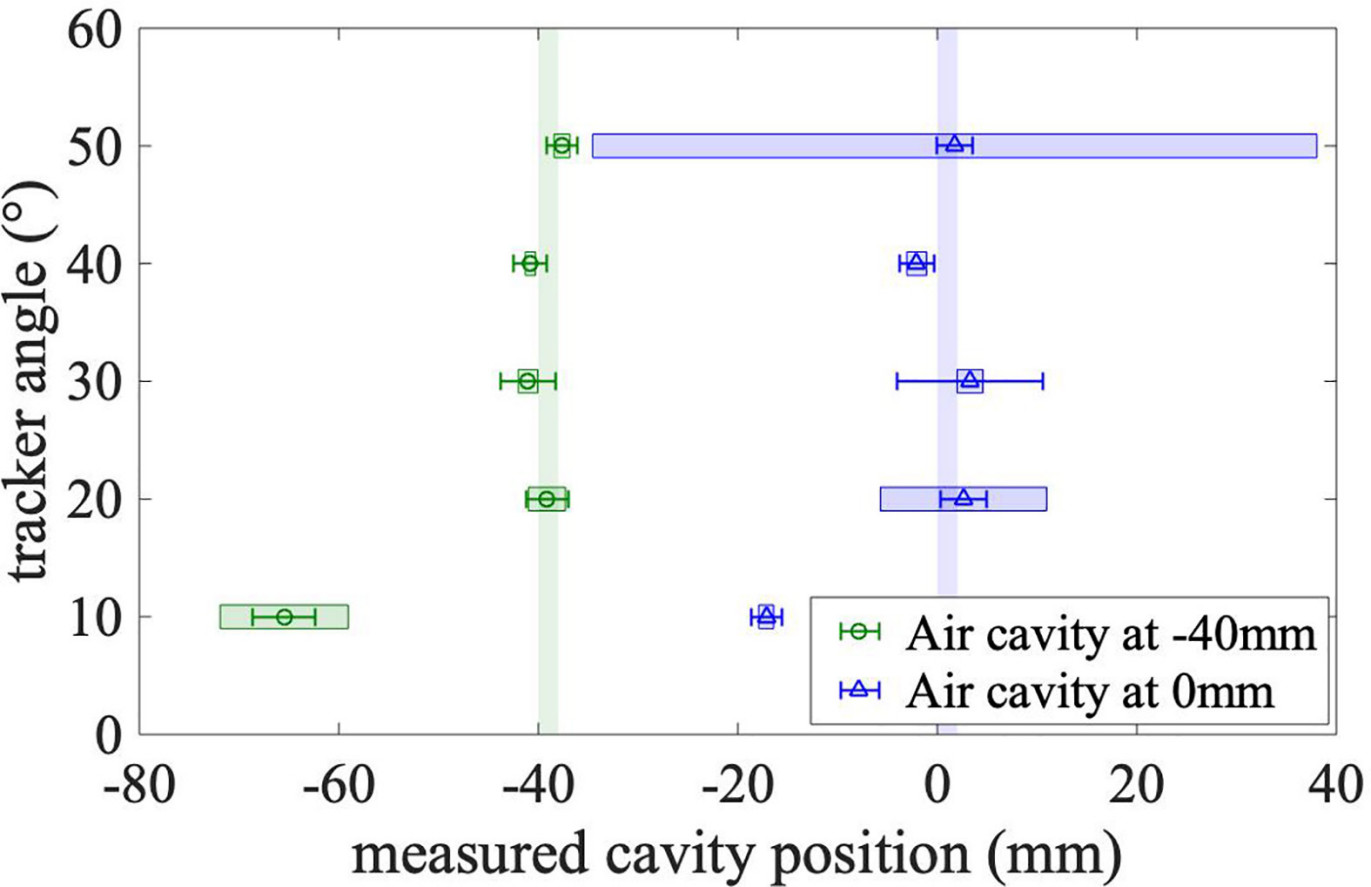
Measured positions of the cavity for different detection angles. The measurement with an air cavity in front of the tumor volume (at -40 mm) is plotted as green circles. The blue triangles show the measurement with the cavity in the middle of the tumor volume (0 mm). The true positions of the air cavity are shown as the vertical green and blue bands. The error bars represent the statistical uncertainty. The colored boxes that are centered on the markers represent the systematic effect of the bin size.

The measured position of the air cavity is defined as the minimum of a parabola drawn through the minimum bin and its both nearest neighbors (see section 2.7.3). **Figure 7** shows the reconstructed positions of the air cavity for two cavity locations and five detection angles. The error bars represent the statistical uncertainty of the minimum of the parabola. For all cavity positions and the mini-tracker positions between 20° and 50°, except for the cavity at 0 mm and the mini-tracker at 40°, the measured cavity position is in agreement with the actual cavity position within the statistical uncertainty of the measurement. It is observed that the localization fails for the smallest investigated detection angle of 10° due to the absence of a clear minimum in the difference of the emission profiles.

In order to quantify the influence of the bin size on the power of the method to localize the air cavity, the localization procedure was conducted for emission profiles with different bin sizes ranging from 3 to 7 mm in steps of 1 mm, besides the 5 mm used previously. The standard deviation of the resulting cavity positions is used as an estimate for the influence of the bin size on the localization. This systematic uncertainty is shown in **Figure 7** as colored error boxes centered on the markers. The localization is found to be more accurate (smaller systematic uncertainties) for air cavities located in front of the tumor. This is expected because more primary carbon ions cross those shallow air cavities, leading to a more pronounced minimum in the difference distribution.

It can be seen that even the influence of the bin size (i.e. the size of the error boxes) cannot explain the observed difference between the measured and the true cavity position at 10°. This difference is probably caused by the geometrical distortion of the emission profile at this small detection angle.

The large systematic uncertainty at a detection angle of 50° for the cavity position at the isocenter can be explained by the ambiguity of the minimum due to the lower number of measured tracks at this detection angle. Depending on the utilized bin size, the position of the minimum is reconstructed either too close to the isocenter or too close to the entrance to the phantom (see also difference plot in **Figure 5E**).

## 4 Discussion

This study employed a mini-tracker made of dead-time-free Timepix3 silicon pixel detectors to measure the charged fragments emerging from a homogeneous PMMA head phantom that is irradiated by a realistic carbon-ion treatment plan with a dose of 3 Gy (RBE). A dataset equivalent to eight mini-trackers with an active area of 2 cm^2^ each was used to detect and localize an air cavity of 2 mm thickness and 80 × 80 mm^2^ transverse area from different detection angles relative to the beam axis. The size of the investigated inter-fractional change is considerably smaller than in previously published studies that used inserts with a thickness of 10 mm (11), 28.5 mm (29) and 28 mm (30).

The suitability of different mini-tracker angles was investigated in terms of detectability and localization of the air cavity.

All investigated measurement settings in terms of the tracker position and the air cavity position resulted in a detectability above the significance threshold of three standard deviations. The larger number of detected secondary ions at small detection angles led to a strongly improved detectability (up to 23 standard deviations) compared with larger detection angles with 4 standard deviations at 40° and above. This shows the large technical potential of the used tracking method for a future use in clinical studies.

The detectability of the air cavity was found to be more significant when the cavity is located in front of the tumor in the path of the primary carbon ions, compared to a cavity located at the center of the tumor. Only cavities located in the primary carbon-ion beam path (in front of the Bragg peak) are relevant for the dose deposition in the tumor and changes in the high-dose-gradient region of the Bragg curve. Therefore, an increased sensitivity to shallower anatomical changes could be an asset of secondary-ion monitoring, leading to a lower number of false-positive detections.

The localization of the air cavity was found to be more accurate at larger detection angles, as expected from geometrical effects in the projection. Air cavities at shallow depths were found to be easier to localize because they cause a more pronounced minimum in the fragment emission profile. A limitation of this localization method is that it will fail if no unambiguous minimum can be identified, which can be the case if the number of measured fragment tracks is too small. The localization could be made more robust by introducing prior knowledge in the analysis, e.g. information about the location of natural cavities from the planning CT.

The proposed analysis uses non-normalized (absolute) fragment emission profiles. This is motivated by the fact that a noticeable difference in the number of measured fragment tracks between two treatment fractions would suggest an important change in the dose to the patient. Concerning the reproducibility of the irradiation, it was found in the presented measurements that the number of detected fragments is relatively constant: a maximum relative difference between repetitions of 0.5% was measured for the smallest number of detected fragments at a detection angle of 50°. Moreover, an analysis of the beam record files of the BAMS showed that the number of primary carbon ions between two irradiations of the same treatment plan has a relative standard deviation of 0.03%, whereas the observed systematic deviation from the number of carbon ions in the treatment plan was 0.4%. It is worth noting that our definition of the detectability takes into account the statistical fluctuation of the number of detected fragments by including a comparison to a no-cavity measurement (see Equation 3).

One aim of this study is to inform the design of a future detection system about the optimum detection angle. It was found that a compromise is to be made between detectability and localization of air cavities. The significance of the cavity detection is maximized at smaller detection angles, while the accuracy of the cavity localization is improved at medium-to-large detection angles. However, taking into account the detectability and localization as well as current hardware limitations (signal pile-up), the optimum detection angle for the presented system was found to be in the range from 20° to 40°. In further studies, a detection system with several mini-trackers placed at different detection angles could allow both parameters to be optimized simultaneously.

All measurements at a specific mini-tracker position were performed without moving the detector or the phantom. Therefore, the data does not include any inter-fractional positioning uncertainty of the mini-tracker. If too large, the positioning uncertainty could impair the performance of the method, leading to a false-positive detection of an inter-fractional anatomical change. Since the magnitude of the positioning uncertainty is determined by the mechanics of the positioning system, the impact of the positioning uncertainty has to be quantified once the final layout of a future detection system is designed. Based on the experience from prompt-gamma range-verification systems, the required positioning reproducibility is expected to be around 1 mm (31).

The observed large values of the detectability show that the effective detection area of 16 cm^2^ is sufficient for the detection of small inter-fractional density changes. The measured number of fragments tracks was also sufficient for the accurate localization of the air cavity. However, the absolute number of detected fragment tracks depends also on the applied dose, the energies of the carbon-ion pencil beams as well as the amount of tissue that has to be crossed by the fragments before reaching the mini-tracker. The setting investigated in this work, where the tumor is located at the center of a head phantom and irradiated by a single field of 3 Gy (RBE), represents a best-case scenario in terms of fragment statistics. Therefore, even larger detection areas would further benefit the performance of the method, leading to smaller statistical uncertainties. Moreover, pointing the mini-tracker axis onto the region of interest in front of the tumor volume could increase the geometrical acceptance and therefore the number of detected fragments.

Future investigations of more realistic cavities in terms of shape, size, position, and composition, might lead to additional information for the geometrical design of future detection systems. Moreover, the size of changes, which can still be detected reliably in a more realistic heterogeneous anthropomorphic head phantom, needs to be investigated. The detection of a potential patient misalignment with respect to the carbon-ion beam (translations and rotations) should be addressed separately.

## 5 Conclusion

The aim of this work was to investigate the power of monitoring carbon ion radiotherapy using tracking of charged nuclear fragments in terms of detection and localization of a 2-mm-thick air cavity in a head-sized PMMA phantom. The performance of the method was analyzed as a function of the position of the mini-tracker. In contrast to previous publications focusing on single pencil beams, a treatment plan with a realistic dose (3 Gy (RBE)), dose rate and tumor size were used. This became possible thanks to the dead-time-free data acquisition of the Timepix3 detector. A dataset equivalent to eight mini-trackers, with an active area of 2 cm^2^ each, was used.

It was found that the presence of the air cavity could be detected for both investigated cavity positions – in the Bragg curve plateau as well as in the center of the target – and for all the investigated detection angles (10° to 50°). The significance of the detection increases with decreasing detection angles – up to 23 standard deviations at 10°. The position of the cavity could be correctly localized within 2 mm, being within the statistical uncertainties, if detection angles ≥ 20° are used. It was found that the air cavity could be localized with higher accuracy and precision if it is located closer to the entrance to the phantom.

The presented results provide important information on the design of future detection systems to be used in clinical trials. Taking into account the ability of the detection system to detect and localize the investigated inter-fractional change, as well as the current hardware limitations, the optimum range of detection angles for the investigated system was found to be between 20° to 30°. The universality of this finding for clinical situations with targets in different depths has to be investigated in the future, ideally within real patient treatments.

## Data Availability Statement

The raw data supporting the conclusions of this article will be made available by the authors, without undue reservation.

## Author Contributions

LG-D and AS planned and performed the measurements, did the data analysis, and wrote the manuscript in the frame of their PhD and Bachelor theses, respectively. LG-D and AS share the first authorship of the manuscript. TG helped design the experiment and provided scientific advice. GE built the experimental setup. RF-B helped perform the measurements. LK helped perform the measurements, design the data analysis, and write the manuscript. MM came up with the original research idea, collected part of the funding, and supervised the scientific rigor of the study. LK and MM share the last authorship of the manuscript. All authors contributed to the article and approved the submitted version.

## Funding

This publication is part of a common project of the DKFZ and HIT, supported by the National Center for Tumor Diseases Heidelberg (NCT). TG was funded by the Deutsche Forschungsgemeinschaft (DFG, German Research Foundation) – Project No.: 426970603.

## Conflict of Interest

The authors declare that the research was conducted in the absence of any commercial or financial relationships that could be construed as a potential conflict of interest.

## Publisher’s Note

All claims expressed in this article are solely those of the authors and do not necessarily represent those of their affiliated organizations, or those of the publisher, the editors and the reviewers. Any product that may be evaluated in this article, or claim that may be made by its manufacturer, is not guaranteed or endorsed by the publisher.

## Footnotes

1 MATLAB, version 9.4.0 (R2018a). The MathWorks, Inc., Natick, Massachusetts, USA
